# MiR-34b-3 and miR-449a inhibit malignant progression of nasopharyngeal carcinoma by targeting lactate dehydrogenase A

**DOI:** 10.18632/oncotarget.10761

**Published:** 2016-07-21

**Authors:** Huiling Li, Xiaoling Li, Xiaolu Ge, Liqing Jia, Zhezhe Zhang, Renpeng Fang, Jing Yang, Jianpin Liu, Shuping Peng, Ming Zhou, Juanjuan Xiang, Zhaoyang Zeng, Wen Zhou, Wei Xiong, Gaoming Xiao, Li Fang, Gui-yuan Li, Zheng Li

**Affiliations:** ^1^ Hunan Cancer Hospital and The Affiliated Cancer Hospital of Xiangya School of Medicine, Central South University, Changsha, China; ^2^ The Key Laboratory of Carcinogenesis of The Chinese Ministry of Health and The Key Laboratory of Carcinogenesis and Cancer Invasion of The Chinese Ministry of Education, Cancer Research Institute, Central South University, Changsha, China; ^3^ High Resolution Mass Spectrometry Laboratory of Advanced Research Center, Central South University, Changsha, China; ^4^ Xiangya School of Medicine, Central South University, Changsha, China

**Keywords:** nasopharyngeal carcinoma, miR-34b-3, miR449a, LC-MS/MS, lactate dehydrogenase A(LDHA)

## Abstract

MicroRNA expression profiling assays have shown that miR-34b/c and miR-449a are down-regulated in nasopharyngeal carcinoma (NPC); however, the targets and functions of miR-34b/c and miR-449a in the pathologenesis of NPC remain elusive. In this study, we verified miR-34b/c and miR-449a were significantly reduced with the advance of NPC. Overexpression of miR-34b-3 and miR-449a suppressed the growth of NPC cells in culture and mouse tumor xenografts. Using tandem mass tags for quantitative labeling and LC-MS/MS analysis to investigate protein changes after restoring expression of miR-34b-3, 251 proteins were found to be down-regulated after miR-34b-3 transfection. Through 3 replicate experiments, we found that miR-34b-3 regulated the expression of 15 potential targeted genes mainly clustered in the key enzymes of glycolysis metabolism, including lactate dehydrogenase A (LDHA). Further investigation revealed that miR-34b-3 and miR-449a negatively regulated LDHA by binding to the 3′ untranslated regions of LDHA. Furthermore, LDHA overexpression rescued the miR-34b-3 and miR-449a induced tumor inhibition effect in CNE2 cells. In addition, miR-34b-3 and miR-449a suppressed LDH activity and reduced LD content, which were directly induced by downregulation of the LDHA. Our findings suggest that miR-34b-3 and miR-449a suppress the development of NPC through regulation of glycolysis via targeting LDHA and may be potential therapeutic targets for the treatment of NPC.

## INTRODUCTION

Nasopharyngeal carcinoma (NPC) is a malignant tumor with poor prognosis. Compared with Western Europe and USA, Southern China region has a high prevalence of 20–50 cases per 100,000 individuals [1]. The majority of NPC patients are diagnosed at late stages and 70% of patients present with cervical lymph node metastasis at first consultation [2]. It is thus important to identify and validate biomarkers for the early diagnosis of NPC.

MicroRNAs (miRNAs) have shown to affect the NPC progression as oncogenes or tumor suppressors [3–7]. MicroRNAs (miRNAs) are short non-coding RNAs of 19–25 nucleotides and induce posttranscriptional silencing mainly by interacting with the 3′untranslated regions (3′UTRs) of target mRNAs [8–9]. A large body of evidence has shown that miRNAs exert an important role in tumorigenesis and drug resistance through the regulation of multiple biological processes including apoptosis, proliferation and metastasis of cancer cells [10–13]. Using a stem-loop RT-PCR or miRNA microarray assay respectively, our previous study and other four independent labs revealed that miR-34b/c and miR-449a are down-regulated in NPC tissues [14–18]. The miR-34 family consists of miR-34a, miR-34b and miR-34c, which are directly regulated by p53 [19–21]. In mammalians, miR-34a is located on chromosome 1p36, while miR-34b and miR-34c are located at chromosome 11q23 and have a common primary transcript [22]. MiR-34b/c has tissue specific functions and different expression patterns in various cancers. Our miRNA microarray assay (GSE32906) [14] has shown that miR-34b/c, but not miR-34a is down-regulated in NPC. MiR-449a is located at 5q11.2 and shares a very similar “seed” sequence and a cohort of targets genes with miR-34b/c [23–26]. In the regulation of spermatogenesis, miR-34b/c and miR-449a have redundant function through targeting of the E2F-Rb pathway [27–28]. However, the precise function and molecular mechanisms of miR-34b/c and miR-449a in the initiation and progression of NPC remain unclear.

In this study we firstly examined the roles of miR-34b/c and miR-449a in the dynamic development of NPC. To clarify the mechanisms of miR-34b/c and miR-449a in the suppression function of NPC, we used tandem mass tags (TMT) isotope labeling technology and LC-MS/MS analysis to explore miR-34b-3 potential target genes. We demonstrated that miR-34b-3 regulated the expression of 15 potential targeted genes including 5 key enzymes in glycolysis metabolism pathway, including lactate dehydrogenase A (LDHA). LDHA promotes a metabolic switch to aerobic glycolysis (Warburg effect), and plays an oncogenic role in most cancers [29–36]. Our results suggest that miR-34b-3 and miR-449a suppress the development of NPC by regulating glycolysis via targeting LDHA.

## RESULTS

### miR-34b/c cluster and miR-449a are down-regulated in NPC

**Figure 1:**
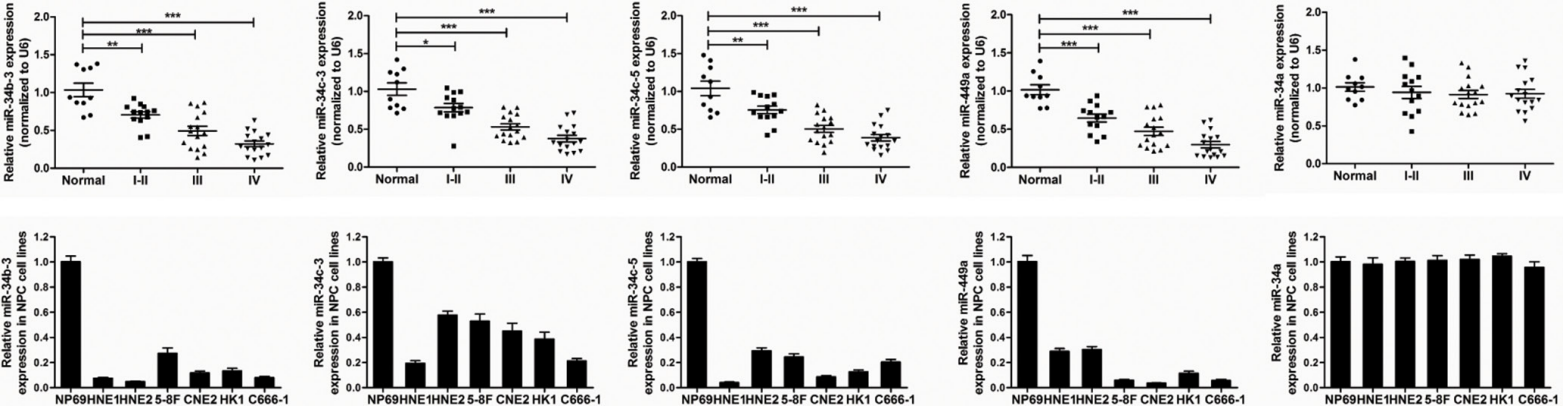
MiR-34b/c cluster and miR-449a are down-regulated in NPC samples and NPC cell lines (Upper line) The real time RT-PCR determination of miR-34 cluster and miR-449a expression in different development stages of NPC (*n* = 45) compared with the non-tumor nasopharyngeal epithelial (normal, *n* = 10). I–II (*n* = 13), III (*n* = 16) or IV (*n* = 16): NPC samples with clinical stage I–II, III or IV disease. Human U6 snRNA was used as an internal control and for normalization of the data. The data are shown as the mean ± SD (*P < 0.05; **P < 0.01; ***P < 0.001, ANOVA test). (Bottom line) The real time RT-PCR was performed to validate the expression of miR-34 cluster and miR-449a in the NPC cell lines and the immortalized normal nasopharynx epithelial NP69 cells. The expression of miRNAs was normalized to U6.

Our previous miRNA array analysis showed that miR-34b/c cluster and miR-449a have low expression in NPC [14]. Here, we further verified the expression levels of miR-34 cluster and miR-449a by RT-PCR in another cohort of NPC samples including 45 NPC tissues of different stages and 10 non-tumor nasopharyngeal epithelial. The clinic information of samples was shown in Supplementary Table S1 (Supplementary information). The expression levels of miR-34b/c cluster and miR-449a were significantly and gradually reduced with advancing stages of NPC, with the lowest expression at the latest stage IV (Figure 1, Upper line; *P < 0.05, **P < 0.01; ***P < 0.001), indicating gradual loss of miR-34b/c cluster and miR-449a expression with the progression of NPC. Compared with the immortalized normal nasopharynx epithelial NP69 cells, miR-34b/c cluster and miR-449a were also significantly down-regulated in NPC cell lines (Figure 1, Bottom line). However, the expression levels of miR-34a were unchanged in NPC tissues and cell lines when compared with non-cancerous nasopharyngeal epithelial tissues and NP69 cells (Figure 1). These data provided strong evidence that miR-34b/c cluster and miR-449a were downregulated in NPC. Since miR-34b-3 and miR-34c-3, miR-34c-5 and miR-449a have the same seed sequences, respectively (Supplementary Table S2), we selected miR-34b-3 and miR-449a as representatives to study the functions and molecular mechanisms at the following study.

### Overexpression of miR-34b-3 and miR-449a suppresses the growth of NPC cells in culture and mouse tumor xenografts

**Figure 2:**
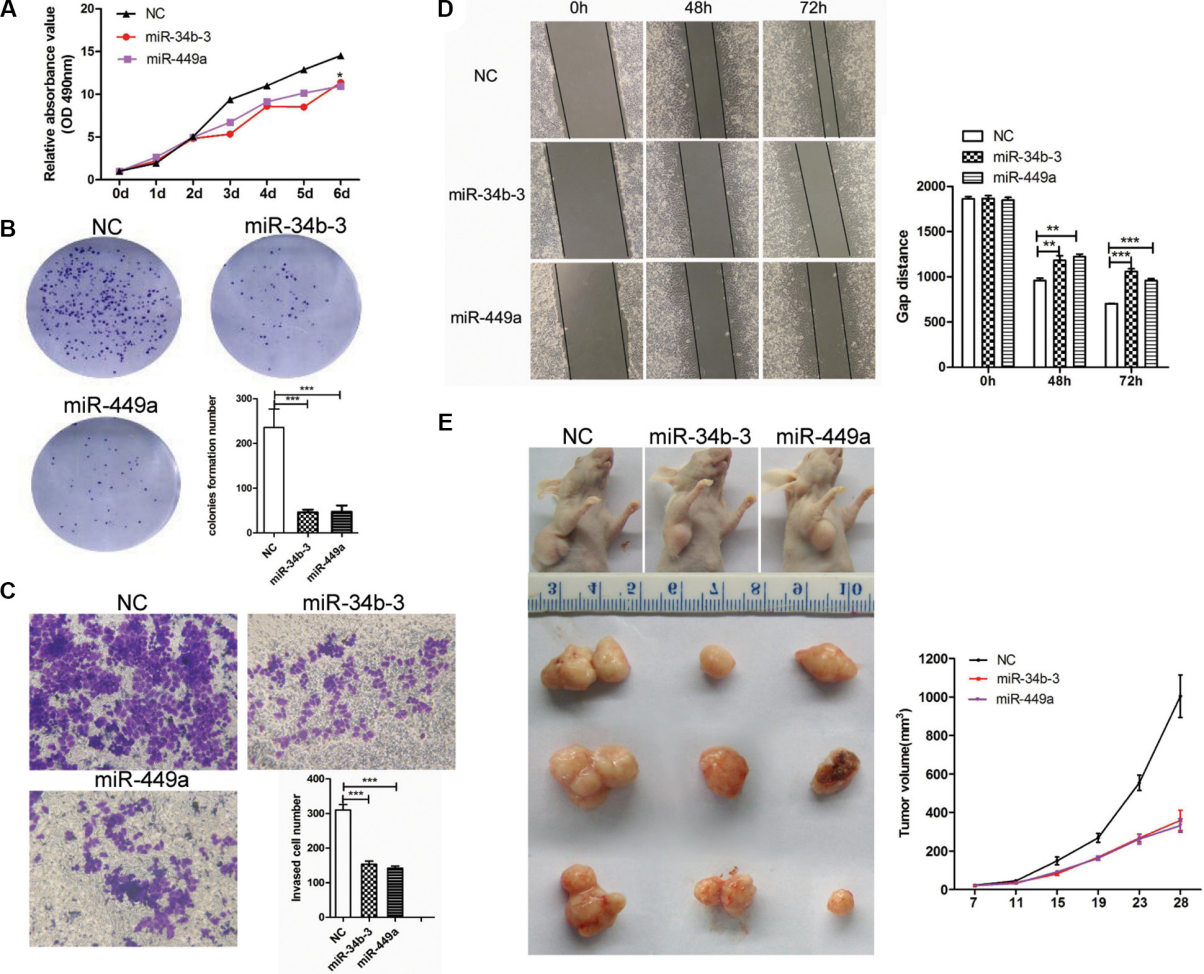
MiR-34b-3 and miR-449a play tumor suppressor roles in NPC (**A**) miR-34b-3 and miR-449a suppressed tumor cell growth. NPC cells CNE2 was transfected with miR-34b-3 mimics, miR-449a mimics or scrambled miRNA (NC) and then subjected to MTT assay. The cell proliferation was measured at the indicated time points. Three independent experiments were performed. The data were shown as the mean±SD (*P < 0.05). (**B**) Colony formation was inhibited in miR-34b-3 and miR-449a over expression CNE2 cells. A total of 1000 cells (CNE2) transfected with miR-34b-3 or miR-449a mimic or scrambled miRNA (NC) were seeded in six-well plates and allowed to grow for 10 days. The clone numbers were quantified. Three independent experiments were performed. The data were shown as the mean ± SD (*P < 0.05; ***P < 0.001). (**C, D**) The wound-healing assay and transwell assay showed that miR-34b-3 and miR-449a inhibited cell motility ability and invasion. The wound gap was measured and migrated stained cells were counted. Three independent experiments were performed and the data were shown as the mean ± SD (**P < 0.01; ***P < 0.001). (**E**) miR-34b-3 and miR-449a suppressed tumor cell growth *in vivo*. Nude mice were subcutaneously injected with transfected CNE2 Cells (1 × 10^6^). The tumors were measured every 4 days, and the growth curves were plotted for each group. The tumor volumes were estimated using the following formula: length × width^2^ × 0.52 (*n* = 5 for each group, ***P < 0.001, ANOVA). All mice were sacrificed 28 days after inoculation and tumors were isolated and photographed at same time point.

To examine the function of miR-34b-3 or miR-449a, NPC cell lines CNE2 and 5–8F were transfected with miR-34b-3 or miR-449a mimics to restore their expression levels. Over-expression of miRNAs was verified by qRT-PCR (Supplementary Figure S1). MTT assay showed that miR-34b-3 and miR-449a overexpression significantly reduced the proliferation of CNE2 cells (Figure 2A, *P < 0.05). Similarly, the colony formation assays verified that miR-34b-3 and miR-449a overexpression resulted in a significantly lower number of colonies than control miRNA (Figure 2B, ***P < 0.001). Additionally, the abilities of invasion of these transfected cells were measured using a transwell assay. Migrated stained cells were counted and statistically analyzed by GraphPad Prism 5 for three independent experiments. Cells transfected with either miR-34b-3 or miR-449a showed significantly reduced cell invasion (Figure 2C; ***P < 0.001). Finally, the migration ability of these transfected cells were measured using a wound-healing assay. The results demonstrated that both miR-34b-3 and miR-449a repressed cell motility. (Figure 2D; **P < 0.01; ***P < 0.001, data from three independent experiments). Similar results were obtained in NPC cell line 5–8F (Supplementary Figure S2). These results clearly demonstrated that overexpression of miR-34b-3 or miR-449a repressed cell proliferation, invasion and migration of NPC cells *in vitro*.

We next asked whether miR-34b-3 or miR-449a overexpression could suppress tumor growth *in vivo*. To this end, CNE2 cells transfected with miR-34b-3 or miR-449a mimics for 48 h were subcutaneously injected into nude mice and tumor formation and volume were monitored. The nude mice receiving cells overexpressing miR-34b-3 or miR-449a formed significantly smaller subcutaneous tumors than mice receiving cells transfected with control miRNA. Moreover, tumor volumes examined at different time points indicated that miR-34b-3 and 449a suppressed tumor growth *in vivo* (Figure 2E).

### TMT quantification labeled protein expression analysis of cells with miR-34b-3 overexpression

**Figure 3:**
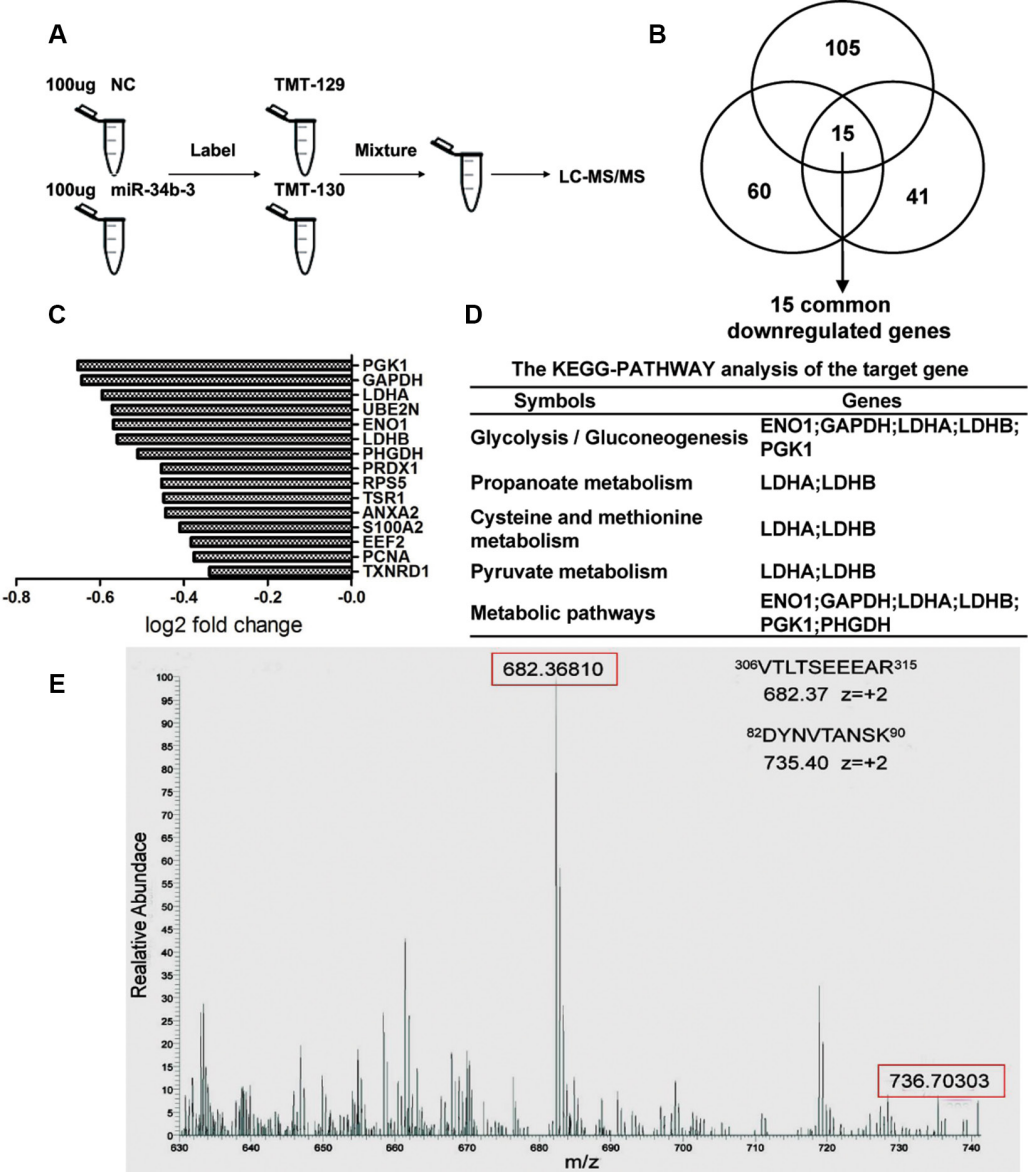
TMT quantification labeled protein expression analysis of cells with miR-34b-3 overexpression (**A**) Samples and schematic outline of the TMT labeling and LC-MS/MS analysis. (**B**) The diagram of three independent experiments of protein expression changes after ectopic miR-34b-3 expression. Proteins with ratio-fold change ≤ 0.8 and log2 fold change ≤ −0.3 between miR-34b-3/NC were considered as potential regulated genes. (**C**) 15 common miR-34b-3 potential targets genes were detected by three biological replicate analysis. The fold changes difference for each gene were shown. (**D**) The potential target genes were analyzed by Kyoto Encyclopedia of Genes and Genomes (KEGG), the top 5 enrichment of biological processes were shown. (**E**) Examples of peptide precursor m/z values and charge states which were identified from the corresponding MS/MS spectrum. LC-MS/MS spectra of precursor ions m/z 682.39 corresponding to residues 306–315 (VTLTSEEEAR) and m/z 735.40 corresponding to residues 82–90 (DYNVTANSK) of LDHA.

To explore the potential target genes of miR-34b-3, we used tandem mass tags (TMT) for quantitative labeling and LC-MS/MS to analyze the different expression of proteins following miR-34b-3 overexpression. CNE2 cells were transiently transfected with miR-34b-3 mimics or negative controls. After 48 h, proteins were extracted from cells and digested. Subsequently, peptides were labeled by TMTs Label Reagents (Thermo, Germany) and mixed to mass spectrometric analysis (Figure 3A). Through analysis of three biological triplicates, we found 251 proteins were down-regulated after ectopic miR-34b-3 expression with ratio-fold change ≤ 0.8 and log2 fold change ≤ −0.3 between miR-34b-3 and NC. Among these 251 proteins, 15 proteins were consistently down-regulated in all three experiments (Figure 3B and 3C). These 15 commonly down-regulated genes were further analyzed by Kyoto Encyclopedia of Genes and Genomes (KEGG). These potential target genes were found to be mainly involved in glycolytic pathways and pyruvate metabolism (Figure 3D). Among these 15 genes, LDHA, LDHB, PGK1 and PHGDH were predicted to have complementary sequences in their 3′UTR with miR-34b/c or miR-449a by one or more Targetscan, Pictar, and miRanda databases. In particular, LDHA was predicted by all three databases. From LC-MS/MS analysis, 18 peptides including 8 unique peptides of LDHA were detected. Figure 3E showed examples of peptide precursor m/z values and charge states, which were identified from the corresponding MS/MS spectrum. LDHA is a key enzyme in glycolysis that plays an oncogene role in the metabolism of tumor cells [29, 37–40]. Increased serum level of lactate dehydrogenase (LDH) is a poor prognostic factor for NPC [41] and also predicts survival in NPC patients treated with palliative chemotherapy [42] or intensity-modulated radiotherapy [43]. Inhibition of LDHA by oxamate is an effective therapeutic strategy for treatment of NPC [44, 45]. Therefore, we focused on LDHA in the following study.

### LDHA is overexpressed in NPC tissues and cell lines

**Figure 4:**
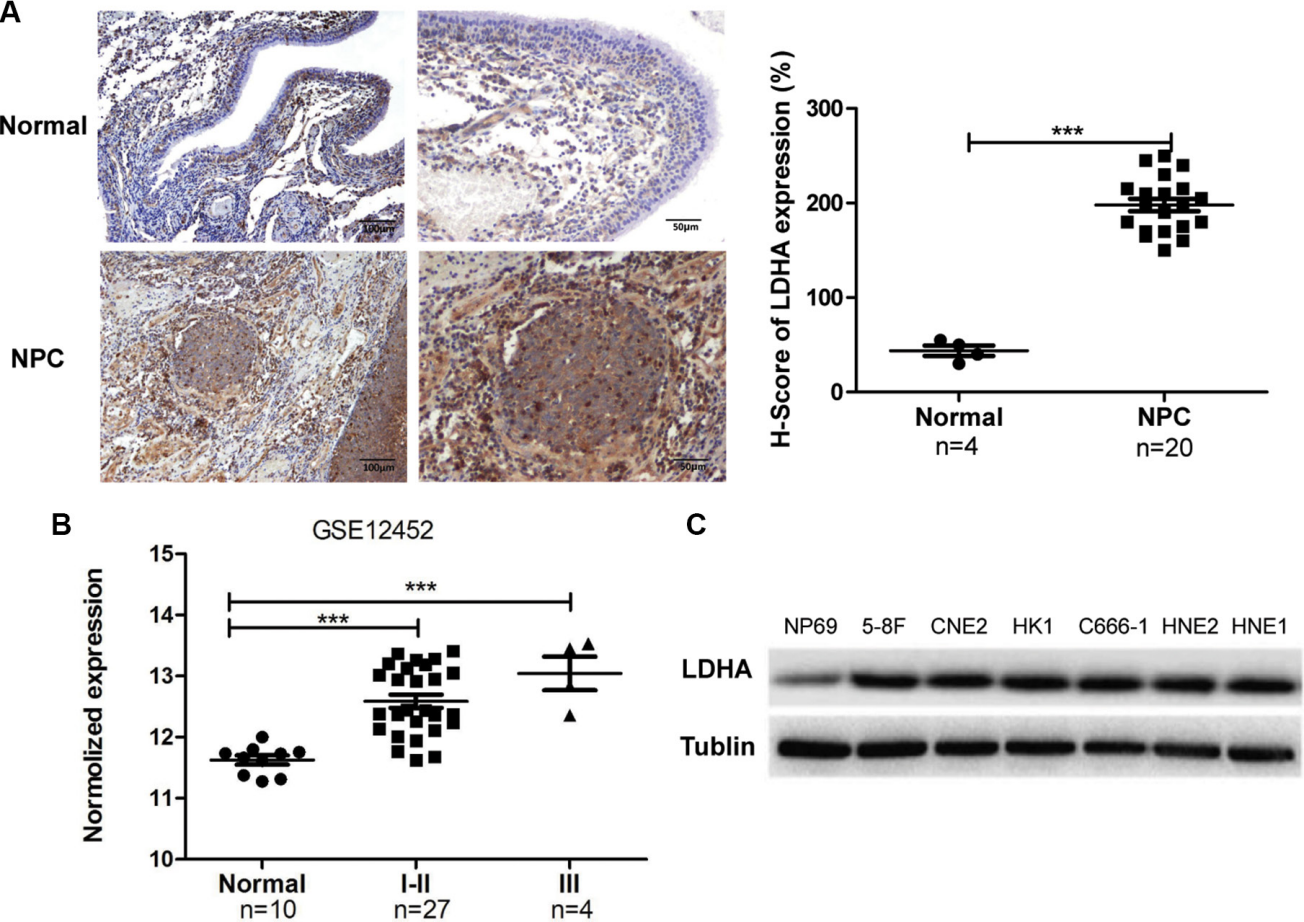
LDHA is overexpressed in NPC tissues and cell lines (**A**) IHC analysis of LDHA protein expression in NPC and non–tumor nasopharyngeal epithelial tissues (normal). Scale bars, 100 μm and 50 μm. Representative images are shown (100 X and 200 X). LDHA expression levels was evaluated in a semiquantitative manner H-score. Each data point corresponds to the LDHA H-score of an individual tissue (***P < 0.001). (**B**) LDHA mRNA expression in different development stages of NPC in the GSE12452 dataset. Normal: non-tumornasopharyngeal epithelial tissues; I–II and III: NPC samples with clinical stage I-II or III disease (***P < 0.001). (**C**) Expression of LDHA was detected by Western blotting in NPC cell lines and immortalized normal nasopharynx epithelial NP69 cells.

To investigate the role of LDHA in the progression of NPC, we detected LDHA expression levels in 20 NPC and 4 non-tumor nasopharyngeal epithelial of paraffin embedded biopsies by immunohistochemistry (IHC). LDHA was overexpressed in NPC tissues (Figure 4A). The clinic information of samples was shown in Supplementary Table S3 (Supplementary information). Furthermore, we analyzed the mRNA expression level of LDHA in 31 NPC samples and 10 non-tumor nasopharyngeal epithelial tissues from GSE12452 database. The results demonstrated that the high mRNA level of LDHA expression was associated with NPC TNM stage (Figure 4B, *P* < 0.001). Further evaluation of the expression levels of LDHA in NPC cell lines by Western blotting verified that LDHA was increased in all NPC cell lines compared with NP69 cells (Figure 4C). These results clearly demonstrated that LDHA was overexpressed in NPC.

### LDHA is a direct target of miR-34b/c and miR-449a

**Figure 5:**
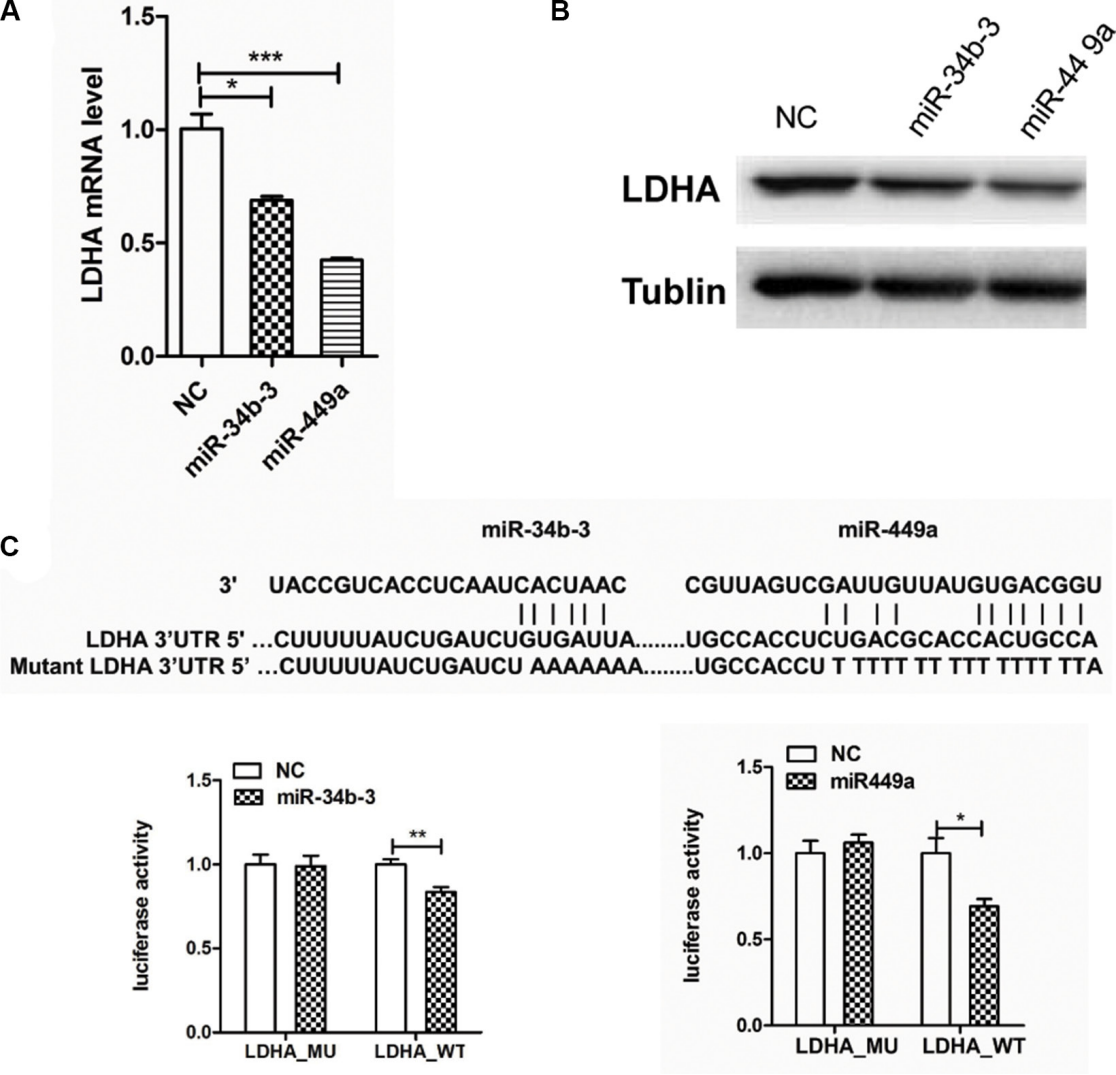
LDHA is a direct target of miR-34b-3 and miR-449a (**A**) miR-34b-3 and miR-449a overexpression reduced LDHA mRNA level. Human β-actin gene was used as an internal control and for normalization of the data. The data are shown as the mean ± SD (*P < 0.05; ***P < 0.001). (**B**) MiR-34b-3 or miR-449a suppressed the protein expression of LDHA. RT-PCR and Western blot analyses of LDHA were performed 48 h after transfection of the same amount of miR-34b-3, miR-449a mimics or scrambled miRNA (NC). (**C**) miR-34b-3 and miR-449a reduced the activity of the luciferase reporter with LDHA wild-type 3′UTR in CNE2 cells, but not that carrying mutated 3′UTR. Sequence alignment of miR-34b-3 and miR-449a and LDHA 3′UTR was shown. The experiments were repeated for three times. The data are shown as the mean ± SD (*P < 0.05; **P < 0.01).

To validate that LDHA is a direct target gene of miR-34b-3 and miR-449a, CNE2 cells were transfected with same amount of miR-34b-3 or miR-449a mimics. After 48 h, the total RNA and protein were extracted for analysis of LDHA mRNA and protein levels. RT-PCR analysis showed that miR-34b-3 and miR-449a overexpression significantly reduced LDHA mRNA level (Figure 5A; *P < 0.05; ***P < 0.001). Western blot analysis showed that the endogenous expression of LDHA protein substantially decreased after miR-34b-3 or miR-449a transfection (Figure 5B). These results indicated that miR-34b-3 and miR-449a over-expression inhibited the endogenous LDHA expression both at mRNA and protein levels.

Furthermore, we cloned the 3′UTR of LDHA downstream of the luciferase open reading frame including wild type or mutant type of miR-34b-3 and miR-449a binding sites. MiR-34b-3, miR-449a mimics or scrambled miRNA were cotransfected with the luciferase constructs. Luciferase activity was detected by Dual-Glo luciferase assay kit (Promega). The results showed significant decrease of luciferase activity in wild-type vector but not in mutant vector after transfection of either miR-34b-3 or miR-449a (Figure 5C; *P < 0.05; **P < 0.001). Furthermore, we found miR-34c-3, miR-34c-5 and miR-34b-5 had binding sites in 3′UTR of LDHA with sequences as predicted by miRanda. Western blot and RT-PCR analysis suggested that miRNAs replenish markedly repressed LDHA protein levels, but the mRNA level of LDHA was unchanged. The luciferase assay demonstrated that these three miRNAs also targeted 3′UTR of LDHA directly (Supplementary Figure S3), suggesting that miR-34c-3, miR-34c-5 and miR-34b-5 are involved in LDHA expression regulation. Collectively, these results clearly indicate that LDHA is a direct target of miR-34b/c cluster and miR-449a.

### MiR-34b-3 and miR-449a inhibit tumor progression in NPC by targeting LDHA to regulate glycolysis

**Figure 6:**
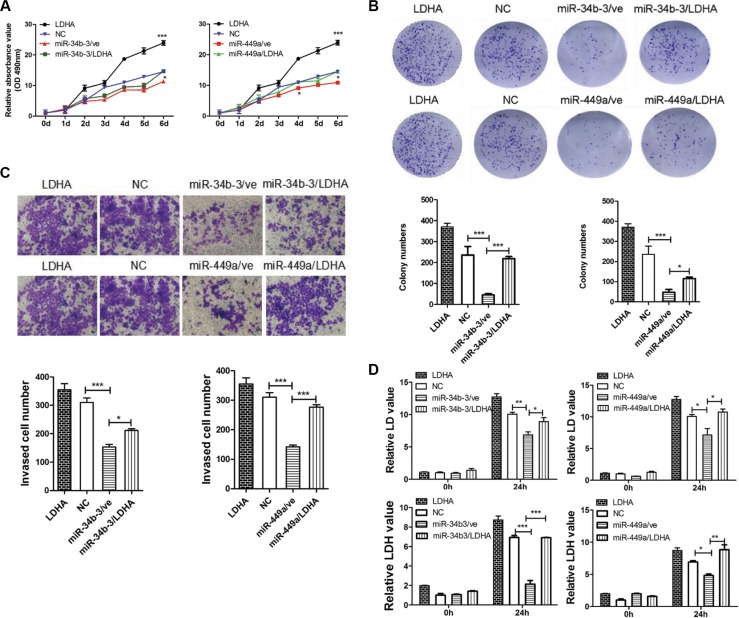
MiR-34b-3 and miR-449a inhibit tumor progression and regulate glycolysis through LDHA in NPC The transfected CNE2 cells were divided into six groups: Cells cotransfected miR-34b-3 with pENTER-LDHA (miR-34b-3/LDHA) or pENTER-3C (miR-34b-3/ve). Cells cotransfected miR-449a mimics with pENTER-LDHA (miR-449a/LDHA) or pENTER-3C (miR-449a/ve). Cells cotransfection of scrambled miRNA and pENTER-3C served as negative control(NC). Cells cotransfection of scrambled miRNA and pENTER-LDHA served as positive control (LDHA). (**A**) MiR-34b-3 and miR-449a inhibit tumor growth through LDHA. Cell proliferation was measured by MTT assay at the indicated time points. Three independent experiments were performed. The data are shown as the mean ± SD (*P < 0.05; **P < 0.001). (**B**) MiR-34b-3 and miR-449a inhibit tumor colony formation through LDHA. The colony formation assay was performed after 10 days culture. The graph summarizes the data from three independent experiments (*P < 0.05; ***P < 0.001). (**C**) MiR-34b-3 and miR-449a inhibit tumor migration through LDHA. The migrated stained cells were counted and statistical analyses were performed using GraphPad Prism 5 from three independent experiments (*P < 0.05; ***P < 0.001). (**D**) MiR-34b-3 and miR-449a regulate glycolysis through LDHA. Cell supernatants at 0 h and 24 h after transfection were collected and tested for the levels of lactic acid (LD) and lactic dehydrogenase (LDH). Three independent experiments were performed (*P < 0.05; **P < 0.01; ***P < 0.001).

We then explored whether the tumor suppressor function of miR-34b-3 and miR-449a depends on LDHA expression. CNE2 cells were cotransfected with miR-34b-3 or miR-449a mimics and the pENTER-LDHA or control plasmid pENTER-3C (ve). Cells cotransfection of scrambled miRNA and pENTER-3C served as negative control (NC). Cells cotransfection of scrambled miRNA and pENTER-LDHA served as positive control (LDHA). Western blot analysis showed that miR-34b-3 and miR-449a mediated decrease of LDHA expression was restored by pENTER-LDHA in CNE2 cells (Supplementary Figure S4). Because pENTER-LDHA vector has C terminal Flag and His tag, the band included ectopic and endogenous LDHA expression. The MTT assay showed that ectopic overexpression of LDHA significantly enhanced the proliferation of CNE2 cells and abolished the suppression of cell proliferation by either miR-34b-3 or miR-449a (Figure 6A; *P < 0.05; **P < 0.001). Similarly, colony formation assay revealed that miR-34b-3 and miR-449a significantly suppressed colony formation compared with negative control, which was significantly prevented by LDHA overexpression (Figure 6B; *P < 0.05; **P < 0.001). Additionally, transwell migration assays showed that cells transfected with miR-34b-3 or miR-449a mimics demonstrated significantly fewer migrated cells than the negative control, which was significantly recovered by LDHA overexpression (Figure 6C, *P < 0.05, **P < 0.01, ***P < 0.001). Collectively, these results demonstrated that LDHA overexpression rescued the suppressive effect of miR-34b-3 and miR-449a in CNE2 cells.

Given LDHA promotes L-lactate and NAD to pyruvate and NADH in the last step of glycolysis pathway, we next assessed whether miR-34b-3 and miR-449a regulated glycolysis in NPC cells through LDHA. CNE2 cells were transfected with miR-34b-3 or miR-449a mimics, and respectively cotransfected with pENTER-LDHA. Subsequently, cell supernatants at 0 h and 24 h were collected and tested for the levels of lactic acid (LD) and lactic dehydrogenase (LDH). The results demonstrated that miR-34b-3 or miR-449a significantly decreased LD content and suppressed LDH activity when compared with negative control, whereas LDHA over-expression rescued the suppressive effect of miR-34b-3 and miR-449a (Figure 6D; *P < 0.05; **P < 0.01; ***P < 0.001, data from three independent experiments). These results suggest that miR-34b-3 and miR-449a execute tumor suppressor gene function in NPC partly due to inhibition of glycolysis by down-regulating LDHA.

## DISCUSSION

In this study, we showed that the expression levels of miR-34b/c cluster and miR-449a were significantly and gradually reduced with advancing stages of NPC, with the lowest expression at stage IV. Consequently, overexpression of miR-34b-3 and miR-449a suppressed the growth of NPC cells in culture and mouse tumor xenografts. Further tandem mass tags isotope labeling and LC-MS/MS analysis revealed that miR-34b/c and miR-449a regulated the expression of 15 potential targeted genes, mainly clustered in key enzymes of glycolysis metabolism, including LDHA. In addition, we found that LDHA was a direct target of miR-34b/c cluster and miR-449a and was overexpressed in NPC. Furthermore, ectopic overexpression of LDHA significantly abolished the suppression of the proliferation, invasion and migration of CNE2 cells by miR-34b-3 or miR-449a. Importantly, miR-34b-3 or miR-449a significantly decreased LD content and suppressed LDH activity, which was restored by ectopic overexpression of LDHA.

From meta-analysis, miR-34a, miR-34b and miR-34c are dysregulated simultaneously in most cancers, such as non-small cell lung cancer, endometrial carcinoma, colorectal cancer, ovarian carcinoma, osteosarcoma [46]. In normal human tissues, miR-34a is universally expressed in most tissues [47]. On the contrary, miR-34b/c is mainly expressed in lung, ovary, testes, and trachea [48], which may explain the obvious reduction of miR-34b/c but not miR-34a in NPC. MiR-449 cluster have very similar sequences and secondary structures belonging to the miR-34 family. Indeed, miR-449a has the same tissue distribution as miR-34b/c and they form a functionally related miRNA family. The double knock out mice of miR-34b/c and miR-449 show basal forebrain structures, absence of motile cilia in trachea and oviducts, and severe disruption of spermatogenesis, but no spontaneous tumor formation [49]. In this study, we found that loss of miR-34b/c and miR-449a was related with the progression of NPC, and overexpression of miR-34b/c and miR-449a inhibited the proliferation, migration and invasion of NPC cells *in vitro* and tumor size *in vivo*. These data suggest an important role for miR-34b/c and miR-449a in the suppression of the tumorigenesis of NPC.

To explore the molecular mechanisms of miR-34b/c and miR-449a as anti-oncogene in NPC, we used quantitative proteomics to find the direct targets of miR-34b/c and miR-449a. Peptides obtained from different experimental conditions are labeled with TMT labeling isobaric mass tags and mixed together. All isobaric mass tags have the same molecular weight in MS1 full mass spectra but produce distinct reporter ions after MS2 that permits peptide quantitation [50, 51]. The relative abundance of a given peptide in each experimental condition can be determined by direct comparison of the reporter ion signal intensities. We compared the differential profiles of proteins between miR-34b-3 overexpressing and negative control cells. Over triplicate experiments, we found that 251 proteins were downregulated after ectopic miR-34b-3 expression, among which 15 proteins were commonly verified in three experiments. In the 15 down-regulated proteins, we noted an overrepresentation of proteins clustered in glycolysis metabolism, propanoate and pyruvate metabolism, suggesting the inhibition of glycolysis may be a major consequence of miR-34b-3 activation.

Among 15 down-regulated proteins, only LDHA was predicted by three database including Targetscan, Pictar, and miRanda. LDHA is a key enzyme at glucose to lactate metabolism and catalyzes the inter-conversion of pyruvate and L-lactate, accompanied with NADH and NAD+ conversion. In 1956, Warburg observed unusual increase of glycolysis rate in cancer cells even under normal oxygen concentrations [52]. Increased LDHA expression promotes the production of ATP, lipids, fatty acids and nucleotides, which are important materials for tumor progression [53–56]. It was reported that increased serum LDH level is poor prognostic factors for NPC [57]. The EBV-encoded latent membrane protein 1 (LMP1) elevates the LDHA activity and lactate production at NPC cells [58]. Our results imply that LDHA has high expression level in NPC tissues and cells. LDHA overexpression promotes NPC cell growth, invasion and lactic acid production.

LDHA plays an important role in tumor maintenance and invasion, and its over expression is associated with tumor proliferation, angiogenesis, metastasis, and resistance to chemotherapy and radiotherapy [59]. It was recently demonstrated that miR-347a directly target LDHA in colorectal cancer cells [60]. MiR-410 represses LDHA expression and promotes embryonic stem cells (hESC) to differentiate into pancreatic endoderm (PE) in gestational diabetes mellitus treatment [61]. In our study, we showed that miR-34b-3 and miR-449a directly targeted LDHA and LDHA overexpression significantly recovered cell proliferation, LD content and LDH activity in NPC cells following transfection of miR-34b-3 and miR-449a. Our findings suggest that miR-34b-3 and miR-449a suppress NPC progression and metastasis partly through inhibition of LDHA.

In conclusion, we verified that miR-34b/c and miR-449a inhibit glycolysis through targeting LDHA in NPC, thereby suppressing the tumor proliferation and progression. These findings will contribute to our understanding of the molecular mechanism by which miR-34b-3 and miR-449a play tumor suppressor roles in the development of NPC and these miRNAs may be potential therapeutic targets to suppress the metabolic reprogramming of NPC cells.

## MATERIALS AND METHODS

### Clinical specimens

Two sets of NPC samples were collected for this study: Set 1, including tissue biopsies of 45 NPC and 10 non-tumor nasopharynx epithelial tissue samples to verify miR-34 and miR-449a expression with qRT-PCR; Set 2, including 20 paraffin-embedded NPC and 4 non-tumor nasopharynx epithelial tissue samples for LDHA detection with IHC. All samples were collected from newly diagnosed NPC patients at the Second Xiangya Hospital (Changsha, China) near two years. All specimens were confirmed by histopathological examination. The patients were informed regarding the sample collection and signed informed consent forms. Collection and use of tissue samples were approved by the Ethical Review Committee of Xiangya Second Hospital. Clinic information was collected from patient medical records and are reported in Supplementary Tables S1 and S3.

### Cell culture and reagents

NPC cell lines HNE2, HNE1, CNE2 5–8F, HK1 and C666-1 were cultured in RPMI-1640 medium supplemented with 10% fetal bovine serum. Immortalized normal nasopharynx epithelial NP69 cells were cultured in RPMI-1640 medium with 10% FBS and growth factors. The cells were grown at 37°C in a humidified atmosphere of 5% CO2. pENTER-LDHA plasmid containing LDHA ORF with C terminal Flag and His tag was obtained from ViGene Biosciences (Jinan, China) LDHA primary antibody was obtained from Proteintech (00012244, Wuhan, China). α-Tublin primary antibody (212144, Wuhan, China) and HRP labeled secondary antibodies were purchased from Vazyme (Nanjjing, China). MiR-34b-3 mimics (MSY0004676, 5′-CAAUCACUAACUCCACUGCCAU-3′), miR-449a mimics (MSY0001541, 5′-UGGCAGUGUAUUGUU AGCUGGU-3′) and scrambled miRNA (a synthesized RNA showing no homology to any human mRNA sequence) were obtained from Qiagen (Valencia, CA, USA). Cells were transfected with miRNA mimics and scrambled miRNA (NC) using Lipofectamine 3000 (Invitrogen), according to the manufacturer's instructions.

### RNA extraction and q-RT-PCR

Total RNA was extracted using Trizol reagent (CWIO, Beijing, China) according to the manufacturer's protocol. The expression level of miR-34b-3 and miR-449a was measured by miRNA primer assays and miScript SYBR^®^ Green PCR Kit (Qiagen) in compliance with manufacturer's instructions. Data were normalized to the expression level of small nuclear RNA RNU6B (U6 snRNA). For LDHA expression analysis, 2 μg total RNA was reverse transcribed into cDNA with AccuRT Genomic DNA Removal kit (Abm, Milton, ON, Canada). qRT-PCR was performed with EvaGreen 2x qPCR MasterMix (Abm) in the CFX96 Real-time PCR Detection System (Bio-Rad, Hercules, CA, USA) to determine the relative expression levels of target genes. The primer used were as follows: LDHA, 5′-TGGAGTGGAATGAATGTTGC-3′ and 5′-ATAGCCCAGGATGTGTAGCC-3′; ACTB (β-actin), 5′-TCACCAACTGGGACGACATG-3′ and 5′-GTCACCG GAGTCCATCACGAT-3′. ACTB was used as the reference and normalization control. The average of three independent analyses for each gene was calculated.

### MTT assay

Cells were transfected with miR-34b-3, miR-449a mimics or scrambled miRNA (NC) for 24 h, and then seeded in a 96-well plate at a density of 2 × 10^3^ cells/well. Changes in cell viability were determined by adding 20 μl of 3-(4,5-dimethylthiazole-2-yl)-2,5-diphenyl tetrazolium bromide (MTT) solution (5 mg/ml) to each well at 0, 1, 2, 3, 4, 5 and 6-day time points. The plate was incubated at 37°C for an additional 4 h. The media was removed and 150 μl of dimethyl sulfoxide was added to each well. The plates were shaken for 10 min to dissolve MTT formazan crystals. The optical density of each well was determined with a scanning multi-well spectrophotometer at a wavelength of 490 nm. The experiments were repeated three times and six parallel samples were measured each time.

### Colony formation assay

Cells were transfected with miR-34b-3, miR-449a mimics or scrambled miRNA (NC) for 24 h, and then seeded in a 6-well plate in triplicate. After incubation for 9–10 days, plates were gently washed with PBS and stained with 0.1% of crystal violet. Colonies with over 50 cells were manually counted. Plating efficiency was calculated by dividing the number of colonies formed in the treated group by that in control.

### Wound healing assay

Cells were transfected with miRNA mimics and grown to 90% confluency in a 6-well plate. A wound was created using a sterile 10 μl pipette tip followed by washing with D-Hanks to remove detached cells. Cells were then cultured in medium with 2% serum. Images were captured 0, 24, 48 and 72 h hours after wounding using a microscope (Nikon).

### Transwell migration assay

Before cell seeding, Corning Costar Transwell 24-well plates (8 μm pores; Corning, NY, USA) were coated with Matrigel (BD) and placed in a cell culture hood for 3 h at 37°C. A total of 3 × 10^4^ cells was seeded in the inserts after transfection and cultured in medium with 2% serum. Normal growth medium was placed in the bottom wells. Cells were then allowed to migrate for 48 h. Migrated cells were fixed with 10% methanol formaldehyde solution for 20 min and allowed to air dry. Invasive cells on the lower surface of the membrane were stained by dipping inserts in a staining solution for 20 min and the stained cells were counted.

### Animal experiments

To confirm the role of miR-34b-3 and miR-449 in the inhibition of cancer cell proliferation *in vivo*, we performed subcutaneous tumor mouse models. Mice were divided into three groups: miR-34b-3, miR-449a and scrambled miRNA(NC). The transfected CNE2 Cells (1 × 10^6^) were washed once with PBS and subcutaneously injected into nude mice (*n* = 5). The tumors were measured every 4 days, and the growth curves were plotted for each group. The tumor volumes were estimated using the following formula: length × width^2^ × 0.52. All mice were sacrificed 28 days after inoculation and tumors were isolated and photographed. All photographed tumors were isolated from single experiments at a similar time points. Animal experiments were approved by the Institutional Animal Care and Use Committee of Central South University (Changsha, China).

### Lactate production assay and LDH activity assay

Lactate production and lactic dehydrogenase (LDH) activity were detected using the Lactate Assay Kit (Jiancheng, Nanjing, China) and LDH assay kit (Jiancheng, Nanjing, China), respectively according the manufacturer's instructions. Results were normalized on the basis of the total protein amount of the cells.

### Protein digestion and TMT labeling

Total protein was extracted using a protein extraction buffer consisting of 7 M urea, 2 M Thiourea, 4% Chaps, 1%DTT, and 0.5% (v/v) protease inhibitor cocktail. The protein concentration was determined by Bradford method using bovine serum albumin as the standard. According to the manufacturer instruction of TMT Isobaric Mass Tag Labeling kit (Thermo, Germany), two samples of miR-34b-3 and negative control (100 μg of each sample) were dissolved in 45 μl of 100 mM triethyl ammonium bicarbonate (TEAB), added with 5 μl 200 mM TCEP and incubated at 55°C for 60 min. 5 μl of 375 mM iodoacetamide (IAA) was added to samples and incubated for 30 min room temperature in the dark. Protein lysates were precipitated with acetone and digested overnight at 37°C with 2.5 μl (1 μg/μl) of Trypsin. Each 100 μg sample was labeled with 41 μl of the TMT Label Reagents. The two-plex TMTs Label Reagents were equilibrated to room temperature and each aliquot was resuspended in 41 μl of anhydrous acetonitrile. Samples were respectively labeled as follows: TMT-129, NC; TMT-130, miR-34b-3. After reaction for 60 min at RT, each tube was added with 8 μl of 5% hydroxylamine and incubated for 15 min. Finally, samples were pooled and desalinated for LC-MS/MS analysis.

### Anion exchange based fractionation of peptides

According to the methods of Wisniewski [62], we used different PH Britton and Robinson buffer to separate our samples. A total of 30 μg peptide mixture was resuspended in pH11 buffer, followed by separation on a pipet-based anion exchanger, which was assembled following the Stage Tip principle [63, 64] by stacking 6 layers of Empore Anion Exchange disk (Agilent technologies, 12145012) and 6 layers of Empore SDB XC disk (Agilent technologies, 12145010) into a 200 μl micropipette tip. For column equilibration and elution of fractions, we used Britton & Robinson buffer composed of 20 mM acetic acid, 20 mM phosphoric acid, and 20 mM boric acid titrated with NaOH to the desired pH. After this, 0.1 ml of methanol was add to the tip-column and centrifuged at 3000rcf/min for 3 min. Peptides were dissolved in 100 μl of PH11 buffer and the flow-through was captured on a StageTip [39] which contained six layers of Anion membrane. Stage tips were washed with 100 μl 0.1% NH4OH in water. Following this, a mixture of 0.1% NH4OH and 80% ACN was used to transfer the peptides to the substrate StageTip. Finally, fractions were subsequently eluted with buffer solutions of pH 11, 8, 6, 5, 4, and 3, respectively.

### LC-MS/MS analysis of peptides

Labeled peptides were analyzed by nano-flow liquid chromatography (Nano-LC)/electrospray ionization (ESI)-tandem MS (MS/MS) using the UltiMate^™^ 3000 RSLCnano system online coupled to an linear trap quadrupole (LTQ)-Orbitrap Velos Pro mass spectrometer (Thermo Fisher Scientific, Bremen, Germany). Peptide mixtures were dissolved in 0.1% formic acid. Separation of peptides was carried out as follows: peptide mixtures were loaded onto one C18 pre-columns (30 μm ×100 mm, Thermo Fisher Scientific, Bremen, Germany) equilibrated with 0.1% (v/v) fluoroacetic acid, washed and pre-concentrated for 5 min at a flow rate of 0.3 μl/min. The pre-column was then switched in line with a C18 RP nano LC column (150 mm × 75 μm, 2 μm, 100Å, Thermo Fisher Scientific, Bremen, Germany) and peptides were eluted with a binary system consisting of solvent A (0.1% formic acid in aqueous phase) and solvent B (0.08% formic acid in 80% ACN) with a flow rate of 0.3 μl/min. The elution linear gradient was as follow: (a) 3% B in 0–5 min, (b) 3–40% B in 5–70 min, (c) 95% B in 75–80 min, (d) 3% B in 81–90 min.

The LTQ-Orbitrap Velos Pro instrument was externally calibrated using LTQ Velos ESI Positive Ion Calibration Solution (Thermo Fisher Scientific, Bremen, Germany). The general mass spectrometric parameters were as follows: spray voltage, 2.2 kV; capillary voltage, 4.5 V; capillary temperature, 250°C; tube lens voltage, 100 V. For data-dependent MS/MS analyses, the software XCalibur (Thermo Fisher Scientific, Bremen, Germany) was used. Full scan MS spectra were acquired at a mass resolution of 60,000 (mass range 100–2000 m/z) in the Orbitrap analyzer.

### Protein identification and quantification

Proteins were identified using Proteome Discoverer 1.4 software (Thermo Scientific, Waltham, MA, USA). Thermo raw files were imported and used to conduct a search of the UniProt KB/Swiss-Prot database (release 2014_10). For database searches, mass tolerances were set to 10 ppm and 0.8 Da for precursor and fragment ions, respectively. Peptides identified with false discovery rates < 1% (*q*-value < 0.01) were discarded. A common contaminants database was also included for quality control. Proteins that met the following criteria were considered differentially expressed proteins: i) proteins were identified based on ≥ 2 peptides with ≥ 95% confidence and ii) proteins were considered decreased clearly when the protein levels demonstrated an averaged ratio-fold change ≤ 0.8 in the LC-MS/MS analyses.

### Bioinformatics analysis

For the convenience of gene annotation, corresponding Entrez gene IDs of the proteins were used for further bioinformatics analysis and these genes were predicted whether have matching sequence with miR-34b/c and miR-449a by 3 common databases such as Targetscan, Pictar, and miRanda. To obtain an overview of the biological functions of the proteins whose expression levels displayed an averaged ratio-fold change ≤ 0.8 difference between the experimental group and control group, we used GSEABASE and GOSTATS of R language software package to analyze the signaling pathways altered after miR-34b-3 restoring.

### Immunohistochemistry

Tissues were fixed with formalin, embedded with paraffin and cut to 4 μm serial section. After deparaffinization and rehydration, tissue sections were incubated in proteinase K for 8 min at room temperature. Slides were incubated in antigen retrieval buffer (0.01 M citrate buffer, PH 6.0) for 30 min, followed by incubation with primary antibody at 4°C overnight. After washing with PBS three times, sections were incubated with polymerized HRP and anti-rabbit IgG for 1 h. The slides were washed and developed using 3′-diaminobenzidine hydrochloride as the chromogen, and counterstained with haematoxylin. After dehydration and mounting, the sections were observed and imaged under a microscope (OLYMPUS BX-51, Japan). Two pathologists independently assessed the immuno-histochemistry in the NPC tissue samples who were blinded to the clinicopathological features and the clinical data. LDHA expression were evaluated in a semiquantitative manner, where by the levels of expression are represented as the percentage of positive cells and the intensity of staining [Hscore = 1 × (% weak) + 2 × (% moderate) + 3 × (% intense) in a range between 0 and 300].

### Western blot analysis

The proteins were extracted using Radio-Immunoprecipitation Assay Buffer (RIPA buffer, Beyotime Biotechnology, Haimen, China). Proteins were quantified using the BCA^™^ Protein Assay Kit (Thermo Fisher Scientific). Equal amount of proteins (50 μg) were separated by 12% sodium dodecyl sulfate-polyacrylamide gel electrophoresis and transferred onto a PVDF membrane. After being blocked with 5% nonfat milk in TBS Tween 20 (TBST; 25 mM Tris pH 7.5, 150 mM NaCl, 0.1% Tween 20) for 1 h at room temperature, membranes were incubated with primary antibodies in 5% nonfat milk in TBST overnight at 4°C. After washing three times with TBST, membranes were then incubated with horseradish peroxidase-labeled secondary antibody for 1 h at room temperature. The signal was visualized using an ECL detection reagent (Vazyme, Nanjing, China) and quantified by using the BioRad ChemiDoc XRS system.

### Luciferase reporter assay

For miRNA target validation, the 3′UTR of LDHA was PCR-amplified using human genomic DNA and cloned into the downstream of pGL3.0 control reporter vector (Promega). Constructs containing mutated miRNA binding sites in 3′UTR were generated using the QuikChange site-directed mutagenesis kit (Stratagene). CNE2 cells were cultured in 24-well plates (1 × 10^5^ cells per well) and co-transfected with 50 nM miRNA mimics, 1 μg firefly luciferase reporter containing the 3′UTR and 100 ng Renilla luciferase reporter. After 48 h, the luciferase activity was measured using the Dual-Glo luciferase^®^ reporter assay system (Promega). Data were normalized against values of co-transfected Renilla luciferase. Cells transfected with scrambled miRNA (a synthesized RNA showing no homology to any human mRNA sequence) served as negative controls. All experiments were performed three times.

### Statistical analysis

Statistical calculations were performed in GraphPad Prism5 software. Student's *t*-tests were used to evaluate significant difference between any two groups of data. One-way ANOVA was used when there are more than two groups. All data are represented as mean ± standard deviation (SD). Difference was considered significant if *p* < 0.05.

## SUPPLEMENTARY MATERIALS




