# Supplementary material for: MiR-34b-3 and miR-449a inhibit malignant progression of nasopharyngeal carcinoma by targeting lactate dehydrogenase A
Source: Oncotarget. 2016 Jul 21;7(34):54838–51. doi: 10.18632/oncotarget.10761 (PMC5342385; doi:10.18632/oncotarget.10761)
Supplement: Supplementary file 1 [file oncotarget-07-54838-s001.pdf]

MiR-34b-3 and miR-449a inhibit malignant progression of nasopharyngeal carcinoma by targeting lactate dehydrogenase A

Supplementary Materials

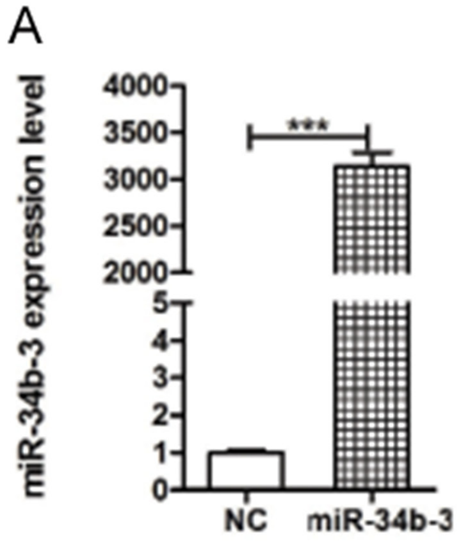

Supplementary Figure S1: The levels of miR-34b-3 and miR-449a in transfected cells. Expression levels of miR-34b-3 and miR-449a in transfected cells were determined by qRT-PCR. Human U6 snRNA was used as an internal control and for normalization of the data. The data are shown as the mean ± SD (****P* < 0.001).

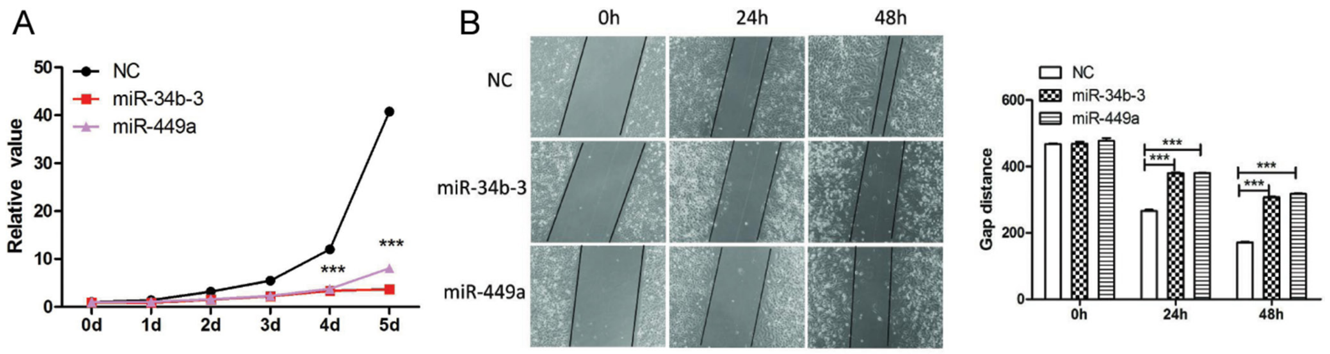

Supplementary Figure S2: MiR-34b-3 and miR-449a play tumor suppressor roles in NPC. (**A**) MiR-34b-3 and miR-449a suppressed tumor cell growth. NPC 5-8F cells were transfected with miR-34b-3 mimics, miR-449a mimics or scrambled miRNA (NC) and then subjected to MTT assay. The cell proliferation was measured at the indicated time points. Three independent experiments were performed. The data are shown as the mean ± SD (****P* < 0.001). (**B**) The wound-healing assay showed that miR-34b-3 and miR-449a inhibited cell motility. Three independent experiments were performed. The wound gap was measured and the data are shown as the mean ± SD (****P* < 0.001).

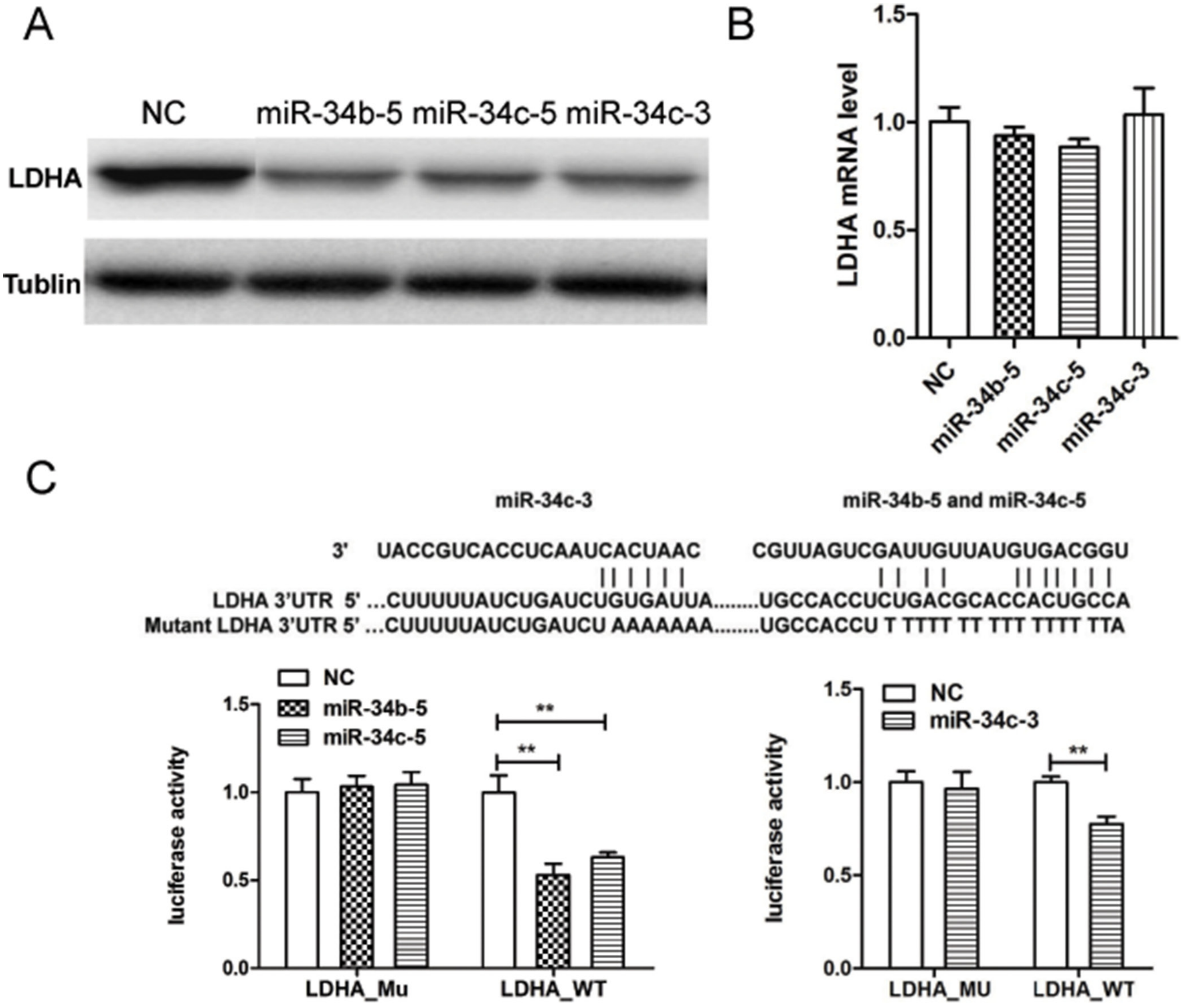

Supplementary Figure S3: LDHA is a direct target of miR-34b-5, miR-34c-5 and miR-34c-3. (**A**) MiRNAs downregulated the protein expression of LDHA. (**B**) MiRNAs overexpression had no affection on LDHA mRNA level. Western blot and RT-PCR analyses of LDHA were performed 48 h after transfection of the same amount of miR-34b-5, miR-34c-5 and miR-34c-3 mimics. (**C**) MiR-34b-5, miR-34c-5 and miR-34c-3 reduced the activity of the luciferase reporter with LDHA wild-type 3′UTR in CNE2 cells, but not that carrying mutated 3′UTR. Sequence alignment of miR-34b-5, miR-34c-5 and miR-34c-3 and LDHA 3′UTR was shown. The experiments were repeated at three times. The data are shown as the mean ± SD (***P* < 0.01).

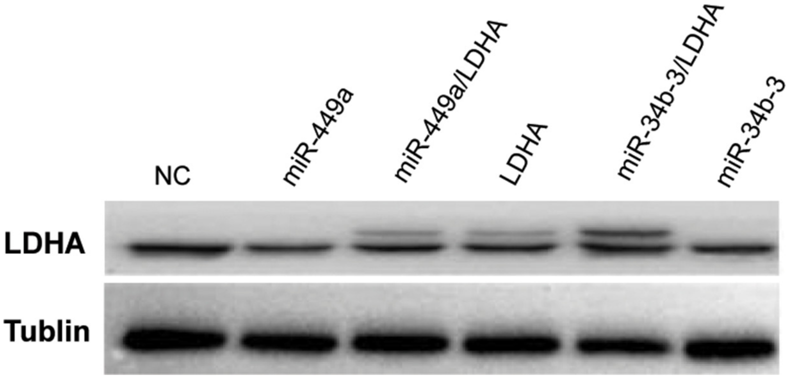

Supplementary Figure S4: miR-34b-3 and miR-449a mediated decrease of LDHA expression was restored by LDHA vector in CNE2 cells. The transfected CNE2 cells were divided into six groups: Cells cotransfected miR-34b-3 with pENTER-LDHA (miR-34b-3/LDHA) or pENTER-3C (miR-34b-3). Cells cotransfected miR-449a mimics with pENTER-LDHA (miR-449a/LDHA) or pENTER-3C (miR-449a). Cells cotransfection of scrambled miRNA and pENTER-3C served as negative control(NC). Cells cotransfection of scrambled miRNA and pENTER-LDHA served as positive control (LDHA). The western blot analysis showed that miR-34b-3 and miR-449a significantly suppressed the endogenous LDHA expression. By contrast, LDHA overexpression rescued the inhibiting effect caused by miR-34b-3 and miR-449a.

Supplementary Table S1: Clinicopathological data of 45 NPC biopsies and 10 non-tumor nasopharyngeal epithelial biopsies for miRNAs expression detection with qRT-PCR. See Supplementary_Table_S1

Supplementary Table S2: The seed sequence of miR34-b/c and miR-449a

miRNA	sequence
hsa-miR-34b-3	CAAUCACUAACUCCACUGCCAU
hsa-miR-34c-3	AAUCACUAACCACACGGCCAGG
hsa-miR-449a	U GGCAGUGU AUUGUUAGCUGGU
hsa-miR-34b-5	U AGGCAGUGUC A UUAGCUGAUUG
hsa-miR-34c-5	A GGCAGUGU AGUUAGCUGAUU GC

MiR-34b-3 and miR-34c-3 have the same seed sequence. MiR-34b-5, miR-34c-5 and miR-449a have the similar seed sequence.

Supplementary Table S3: Clinic information for the 20 paraffin-embedded NPC and 4 non-tumor nasopharyngeal epithelial biopsies for LDHA detection with IHC

Samples No.	Gender (M = Male F = Female)	Age at Diagnosis	TMN	Clinic stages
S01	M	52		
S02	M	38		
S03	F	49		
S04	M	47		
S05	M	47	T2N2M0	III
S06	F	52	T2N1M0	II
S07	M	62	T3N1M0	III
S08	M	48	T2N1M0	II
S09	M	64	T2N3M0	IV
S10	F	50	T1N1M0	II
S11	M	43	T4N1M0	IV
S12	M	44	T2N0M0	II
S13	M	47	T2N2M0	III
S14	M	39	T2N2M0	III
S15	F	52	T1N0M0	I
S16	M	51	T2N1M0	II
S17	F	36	T2N1M0	II
S18	M	39	T3N1M0	III
S19	M	57	T3N2M0	III
S20	M	62	T2N3M0	IV
S21	M	59	T2N2M0	III
S22	F	56	T2N2M0	III
S23	M	43	T1N1M0	II
S24	M	56	T2N2M0	III
