# Supplementary material for: MiR-34b-3 and miR-449a inhibit malignant progression of nasopharyngeal carcinoma by targeting lactate dehydrogenase A
Source: Oncotarget. 2016 Jul 21;7(34):54838–51. doi: 10.18632/oncotarget.10761 (PMC5342385; doi:10.18632/oncotarget.10761)
Supplement: Supplementary file 2 [file oncotarget-07-54838-s002.docx]

Table S1 Clinic information for the 45 primer NPC biopsies and 10 non-tumor nasopharyngeal epithelial (normal) used for miRNA expression detection with qRT-PCR

| Samples No. | Gender (M=Male F=Female) | Age at Diagnosis | TMN | Clinic stages |
| --- | --- | --- | --- | --- |
| S01 | M | 35 |  |  |
| S02 | F | 41 |  |  |
| S03 | M | 52 |  |  |
| S04 | M | 37 |  |  |
| S05 | F | 45 |  |  |
| S06 | M | 53 |  |  |
| S07 | M | 49 |  |  |
| S08 | M | 36 |  |  |
| S09 | M | 56 |  |  |
| S10 | F | 41 |  |  |
| S11 | F | 32 | T3N1M0 | III |
| S12 | M | 49 | T2N1M0 | II |
| S13 | F | 53 | T1N3M1 | IV |
| S14 | F | 59 | T2N1M0 | III |
| S15 | M | 62 | T1N0M0 | I |
| S16 | M | 63 | T2N2M0 | III |
| S17 | M | 38 | T3N1M0 | III |
| S18 | M | 67 | T2N1M0 | II |
| S19 | F | 70 | T1N1M0 | II |
| S20 | M | 33 | T2N3M0 | IV |
| S21 | M | 44 | T4N0M0 | IV |
| S22 | M | 49 | T1N1M0 | II |
| S23 | M | 46 | T2N2M0 | III |
| S24 | M | 31 | T4N1M0 | IV |
| S25 | F | 47 | T2N3M0 | IV |
| S26 | F | 52 | T2N0M0 | II |
| S27 | M | 58 | T2N1M0 | II |
| S28 | M | 63 | T2N2M0 | III |
| S29 | M | 43 | T2N2M0 | III |
| S30 | M | 51 | T2N2M0 | III |
| S31 | F | 36 | T1N0M0 | I |
| S32 | F | 29 | T1N1M0 | II |
| S33 | M | 37 | T1N3M1 | IV |
| S34 | M | 56 | T3N1M0 | III |
| S35 | M | 59 | T4N1M0 | IV |
| S36 | M | 62 | T4N0M0 | IV |
| S37 | M | 45 | T2N1M0 | II |
| S38 | F | 46 | T2N1M0 | II |
| S39 | M | 53 | T4N1M0 | IV |
| S40 | F | 62 | T3N1M0 | III |
| S41 | M | 51 | T4N2M0 | IV |
| S42 | M | 38 | T3N2M0 | III |
| S43 | M | 46 | T2N3M0 | IV |
| S44 | F | 58 | T2N2M0 | III |
| S45 | M | 56 | T2N2M0 | III |
| S46 | M | 51 | T2N3M0 | IV |
| S47 | M | 36 | T4N1M0 | IV |
| S48 | F | 59 | T2N3M0 | IV |
| S49 | F | 64 | T3N1M0 | III |
| S50 | M | 47 | T1N1M0 | II |
| S51 | M | 49 | T2N1M0 | II |
| S52 | M | 56 | T2N2M0 | III |
| S53 | M | 52 | T2N3M0 | IV |
| S54 | M | 43 | T3N1M0 | III |
| S55 | M | 51 | T2N3M0 | IV |
